# Incremental Value of CSF Biomarkers in Clinically Diagnosed AD and Non-AD Dementia

**DOI:** 10.3389/fneur.2020.00560

**Published:** 2020-06-25

**Authors:** Jean-Baptiste Oudart, Zoubir Djerada, Vignon Nonnonhou, Sarah Badr, Laurie-Anne Bertholon, Anis Dammak, Yacine Jaidi, Jean-Luc Novella, Nicolas Pallet, Philippe Gillery, Rachid Mahmoudi

**Affiliations:** ^1^Laboratory of Biochemistry and Molecular Biology, Faculty of Medicine, University of Reims Champagne-Ardenne, SFR CAP-Santé (FED 4231), Reims, France; ^2^CNRS UMR 7369, Matrice Extracellulaire et Dynamique Cellulaire - MEDyC, Reims, France; ^3^Laboratory of Biochemistry, Pharmacology and Toxicology, Reims University Hospital, Reims, France; ^4^Department of Pharmacology, E.A.3801, SFR CAP-santé, Reims University Hospital, Reims, France; ^5^Champagne-Ardenne Resource and Research Memory Center (CMRR), Maison Blanche Hospital, Reims University Hospital, Reims, France; ^6^Department of Internal Medicine and Geriatrics, Maison Blanche Hospital, Reims University Hospital, Reims, France; ^7^Department of Psychiatry, Public Institution of Mental Health Marne, Châlons-en-Champagne Cedex, France; ^8^Faculty of Medicine, University of Reims Champagne-Ardenne, Reims, France; ^9^Department of Biochemistry, Georges Pompidou European Hospital, Assistance Publique - Hôpitaux de Paris, Paris, France

**Keywords:** Alzheimer disease, CSF biomarkers, amyloid peptide, cut-off values, diagnostic algorithm

## Abstract

**Background:** Cerebrospinal fluid (CSF) biomarkers are used to diagnose Alzheimer disease (AD), especially in atypical clinical presentations. No consensus currently exists regarding cut-off values. This study aimed, firstly, to define optimal cut-off values for CSF biomarkers, and secondly, to investigate the most relevant diagnostic strategy for AD based on CSF biomarker combinations.

**Methods:** A total of 380 patients were prospectively included: 140 with AD, 240 with various neurological diagnoses (non-AD). CSF biomarkers were measured using ELISA. Univariate and multivariate analyses were performed using random forest and logistic regression approaches.

**Results:** Univariate receiver operating curve curves analysis of T-Tau, P-Tau_181_, Aβ_42_, Aβ_40_ concentrations, and Aβ_42_/Aβ_40_ ratio levels showed AD cut-off values of ≥355, ≥57, ≤706, ≥10,854, and ≤0.059 ng/L, respectively. Multivariate analysis using random forest and logistic regression found that the algorithm based on P-Tau_181_, Aβ_42_ concentrations and Aβ_42_/Aβ_40_ ratio yielded the best discrimination between AD and non-AD populations. The cross-validation technique of the final model showed a mean accuracy of 0.85 and a mean AUC of 0.89.

**Conclusion:** This study confirms that the Aβ_42_/Aβ_40_ ratio was more useful than the Aβ_40_ concentration in discriminating AD from non-AD populations in daily practice. These results indicate that the Aβ_42_/Aβ_40_ ratio should be assessed in all cases, independently of Aβ_42_ concentrations.

## Introduction

Alzheimer disease (AD), the most common cause of dementia, is a progressive neurodegenerative disease clinically characterized by memory impairment and/or deficit in other cognitive domains associated with functional decline (1). For many years, the diagnosis of AD remained probabilistic and was based on clinical features associated with neuropsychological testing and neuroimaging (2). These old criteria were useful at the stage of dementia and were defined as cognitive impairment including memory impairment impeding daily life activities and interactions with the social network. This clinical diagnostic method led to a diagnosis of “probable” and “possible” AD, based on different levels of clinical confidence. Despite a lack of specificity (70%), this method has relatively good sensitivity (80%) (3). To increase diagnostic accuracy, especially in case of atypical clinical presentation, the International Working Group (IWG) recommendations proposed the use of diagnostic biomarkers such as neuroimaging (e.g., 8F-fluorodeoxyglucose PET, volumetric MRI and amyloid PET) and/or cerebrospinal fluid (CSF) biomarker assessment (4). The association of clinical criteria of AD with CSF biomarkers clearly improved diagnostic accuracy, raising it to over 80% (5). CSF biomarkers could also be useful for discriminating AD from other dementias [e.g., dementia with Lewy bodies (DLB)] (6).

Core CSF biomarker assessment is defined as a combination of amyloid-β 1-42 peptide (Aβ_42_, which is correlated with APP metabolism and amyloid deposition), Total Tau protein (T-Tau) which reflects neurodegeneration, and phosphorylated Tau protein (P-Tau_181_) which reflects tangle pathology measurement (7). According to the literature, these core biomarkers have a high specificity and sensitivity for discriminating AD from other dementias (8). The typical CSF biomarker profile in AD associates increased T-Tau and P-Tau_181_ concentrations and decreased Aβ_42_ peptide concentration. As misclassifications may be due to inter-individual variability in overall amyloid peptide production, amyloid-β 1-40 peptide (Aβ_40_) assay, which closely reflects total amyloid load in the brain, has more recently been validated and implemented in the diagnostic sequence (9).

However, no consensual cut-off values have been established at this time, except for P-Tau_181_ with a cut-off of around 60 ng/L (10). The optimal cut-off value for Aβ_40_ peptide has not been widely studied, although increased concentrations have been reported in AD compared with non-AD populations (11). Hence, most clinical studies are based on *ad hoc* optimum cut-off definitions. Differences are mostly reported for amyloid peptide measurement (*i.e*. Aβ_42_ peptide cut-off values may range from 550 to 800 ng/L, even when using the same ELISA method) (12). It is widely acknowledged that pre-analytical factors play a key role in this variability, but are not sufficient to wholly explain these discrepancies. To significantly reduce the substantial variability in Aβ_42_ measurements across laboratories, three Certified Reference Materials (CRMs) for the measurement of Aβ_42_ peptide have been produced by the International Federation of Clinical Chemistry and Laboratory Medicine (IFCC) and the Joint Research Centre (JRC), and are now available.

It has been clearly demonstrated that a combination of CSF biomarkers that includes Aβ_42_/Aβ_40_ ratio calculation, significantly improves the discriminatory capacity in the diagnosis of AD (13, 14). Nevertheless, the added value of CSF Aβ_40_ concentration in the classification strategy of AD diagnosis remains controversial (11, 15). Different classification strategies involving CSF biomarkers are currently used. For instance, CSF Aβ_40_ peptide assessment may be performed together with the core biomarkers (i.e., T-Tau, P-Tau_181_ and Aβ_42_) in all patients, or may only be performed in a second stage, after assessment of the three core biomarkers, especially in case of conflicting results. However, there is no consensus as to the combination of CSF biomarker that has the highest sensitivity and specificity for the diagnosis of AD. A recent study concluded that the CSF Aβ_42_/Aβ_40_ ratio should be calculated in all patients, independently of Aβ_42_ concentration, to improve AD diagnosis (16).

To this end, we undertook the present study firstly, to define optimal cut-off values for CSF biomarkers, and secondly, to evaluate the incremental value of Aβ_40_ concentration or Aβ_42_/Aβ_40_ ratio compared with the standard classification strategy of core CSF biomarkers. The secondary objective was to identify the diagnostic algorithm that was best able to distinguish AD patients from other dementias.

## Materials and Methods

### Study Design

This was a single-centre, prospective study to evaluate the incremental value of CSF biomarker combinations in the diagnosis of AD. An “AD profile” was defined as increased CSF concentrations of T-Tau and P-Tau_181_ associated with decreased Aβ_42_ peptide concentrations, and/or a decreased Aβ_42_/Aβ_40_ ratio. As previously described, an increased concentration of Aβ_40_ peptide was also considered a criterion compatible with an AD profile (13). In contrast, a “Normal profile” was considered if CSF concentrations of T-Tau, P-Tau_181_, Aβ_42_, Aβ_40_ and Aβ_42_/Aβ_40_ ratio were within the optimal cut-off values identified by ROC curve analysis on our sample. Isolated decreased CSF Aβ_42_ peptide concentration associated with an Aβ_42_/Aβ_40_ ratio within the cut-off value was also considered as a normal profile.

### Clinical Enrollment

This study included patients from the memory consultations or geriatric medicine units of Reims University Hospital (Reims, France) between January 2011 and September 2016. Subjects who were not affiliated to any social security regime, as well as subjects under legal guardianship were excluded. All included patients underwent baseline physical and neurological examination associated with neuropsychological evaluation including Mini-Mental State Examination (MMSE), brain imaging and CSF biological measurements. Patients were monitored until the end of the study by clinical examinations. Clinical, neuropsychological and imaging data were reviewed by an independent board (multidisciplinary team including neurologist, geriatrician, and psychiatrist), from baseline to the end of follow-up, for a final diagnosis, but the board members were blinded to the results of CSF biomarker assays.

A total of 380 patients were sequentially included in this study: 140 patients with AD and 240 patients with various non-AD diagnoses (i.e., dementia with Lewy bodies (DLB) (*n* = 12), frontotemporal lobe degeneration (*n* = 14), vascular dementia (*n* = 42), psychiatric disorder (*n* = 31), mixed neurodegenerative disease (*n* = 70), composed of a combination of two or more concomitant neurodegenerative disorders, including AD). For these patients, AD was not mainly involved in the retained diagnosis from the multidisciplinary team. All AD patients met the diagnostic criteria for probable AD according to the Diagnostic and Statistical Manual of Mental Disorders, fourth edition (DSM IV) (17), and the criteria of the National Institute of Neurological and Communicative Disorders and Stroke and the Alzheimer's disease and Related Disorders Association (NINCDS-ADRDA) (2). For patients with other types of dementia (non-AD population), the diagnoses were made according to specific criteria: diagnosis of DLB was made according to the DLB Consortium criteria (18), frontotemporal lobe degeneration was diagnosed according to the criteria described by Neary et al. (19), and Rascovsky et al. (20). Vascular dementia was diagnosed according to the NINDS-AIREN criteria (21). Patients with psychiatric disorders, such as bipolar disorder, depression and anxiety were diagnosed according to DSM IV criteria (17). The study was approved by the regional ethics committee (CPP Est III; protocol number 2014-A00056-41), and written informed consent was obtained from each participant and their main caregiver.

### CSF Analysis

Lumbar puncture was performed in the sitting position according to standardized procedures. All CSF samples were collected in 10 mL polypropylene tubes (ref. 62.610.201, Sarstedt, Nümbrecht, Germany) and transported to the laboratory within 2 h. Samples were then centrifuged for 10 min at 2,000 *g* at 4°C, transferred to another polypropylene tube and immediately stored at −80°C until analysis. CSF T-Tau, P-Tau_181_, Aβ_42_, and Aβ_40_ peptide concentrations were measured using commercially available sandwich ELISAs (INNOTEST, Fujirebio Europe, Ghent, Belgium) according to the manufacturer's instructions. Biomarkers were included in routine clinical diagnosis runs of the laboratory, which is part of an external quality control program for the Alzheimer's Association. CSF analyses were performed blinded to the clinical diagnoses.

### Statistical Analysis

Statistical analyses were performed with R 3.1.4 (The R Foundation for Statistical Computing, http://www.r-project.org). Continuous variables are described as mean and standard deviation (SD) and categorical variables as number (percentage). A sample size of 380 patients including 140 cases and 240 controls provided >80% power to detect a minimal area under the receiver operating characteristics (ROC) curve (AUC) of 0.583 compared to the null hypothesis of an AUC of 0.50 (equivalent to no diagnostic value) using a 2-sided test at a significance level of 0.05. This sample size also provided at least 80% power to detect a 0.13 unit difference between a statistical model with an AUC of 0.58 and another statistical model with an AUC of 0.72 using a 2-sided *z*-test at the significance level α = 0.05. The normality of distributions was assessed using the Shapiro–Wilk and Kolmogorov–Smirnov tests. Non-parametric univariate analyses were done for continuous variables using the Mann–Whitney test. Categorical variables were assessed using the chi-square test, or two-tailed Fisher's exact tests when the expected number in any cell was <5.

To assess the discriminatory capacity of univariate biomarkers, a ROC curve was created, and the AUC was calculated. The cut-off values were estimated using the Youden index (22). The multivariate analysis was performed by a random forest algorithm and by binary logistic regression models in order to define the best diagnostic algorithm for the discrimination between AD and non-AD populations. The biomarkers with greatest discriminatory capacity were identified by the Gini-Index using random forest algorithms (random Forest package R) (23). This procedure is considered a standard non-parametric classification for constructing prediction rules without making any prior assumptions as to the form of their association with the outcome. The hyperparameters and tuning strategies for random forest were performed as described using the tune Ranger package and were as follows: number thread/processor = 2, number of predictors sampled for splitting at each node = 5, minimum size of terminal nodes = 27, sample fraction = 0.202, number of trees to grow = 1000, respect.unordered.factors = order, a sampling of cases done with replacement (24). Others hyperparameters were set to their default value.

Binary logistic regression was applied to calculate the probability of combined biomarkers for the diagnosis of AD (25, 26). CSF concentrations of T-Tau, P-Tau_181_, Aβ_42_, Aβ_40_ and Aβ_42_/Aβ_40_ ratio as biomarkers were chosen by fitting a logistic regression model using forward/backward stepwise selection. Akaike information criterion (27) and Bayes information criterion (28) were used to find good candidate models for biomarker combinations. The goodness-of-fit and appropriateness of the logistic regression model were evaluated using the Nagelkerke R squared values and Hosmer–Lemeshow value, by the overall correct percentage of classification, absolute standardized residuals, QQplot or residual, residual vs. covariate and by residual vs. predicted value. Multicollinearity was checked for all analyses and the Wald test was used for hypothesis testing. The stability and robustness of the final model and its estimates were validated using bootstrap resampling (*n* = 1000). ROC curves for combined biomarkers were constructed using the predictive probability as a covariate.

The performance of the final model was validated using the cross-validation technique (29, 30). To this end, we randomly split the data into twenty different estimations (or training) sets and twenty different test (or validation) sets. Each estimation set consisted of data from 95% of patients; the corresponding test set contained the data from the remaining 5% of patients. The final model was fitted to each of the estimation data sets and then the parameter estimates were used to predict the observed class for each test set. The accuracy (1-misclassification error or the proportion of correctly classified patient's i.e., the sum of true positive and true negative tests) and the AUC of ROC curves were assessed using pooled observed and predicted class.

## Results

Demographic, clinical and biological characteristics of the study population are summarized in Table 1. T-Tau and P-Tau_181_ concentrations were significantly higher in the AD population than in the non-AD population (617 and 82 ng/L vs. 319 and 47 ng/L, *p* < 0.001, respectively) whereas CSF Aβ_42_ concentration and Aβ_42_/Aβ_40_ ratio were significantly lower in AD vs. non-AD patients (513 ng/L and 0.044 vs. 786 ng/L and 0.077, *p* < 0.001, respectively).

**Table 1:** Characteristics of the study population.

		**AD**	**Non-AD**
Gender	*n*	140	240
	M/F	87/53^*^	113/127
Age (years)	Mean (SD)	74.6 (9.4)	75.5 (10.0)
	Min/max	54/92	44/94
MMSE score	Mean (SD)	18.6 (6.1)^**^	20.5 (5.9)
	Min/max	4/29	1/30
T-Tau (ng/L)	Mean (SD)	617 (354.4)^***^	319 (247.7)
	Min/max	86/2312	72/2338
P-Tau_181_ (ng/L)	Mean (SD)	82 (35.5)^***^	47 (22.3)
	Min/max	17/258	15/153
Aβ_42_ (ng/L)	Mean (SD)	513 (238.3)^***^	786 (350.2)
	Min/max	87/1635	87/2065
Aβ_40_ (ng/L)	Mean (SD)	12 956 (5921.3)^**^	11 304 (5370.9)
	Min/max	1219/30682	1608/29063
Aβ_42_/Aβ_40_ ratio	Mean (SD)	0.044 (0.020)^***^	0.077 (0.034)
	Min/max	0.014/0.15	0.021/0.217

*AD Alzheimer Disease, non-AD various non-AD diagnoses, MMSE Mini Mental State Examination*.

* *p < 0.05*,

** *p < 0.01*,

*** *p < 0.001 compared to non-AD*.

**Figure 1:**
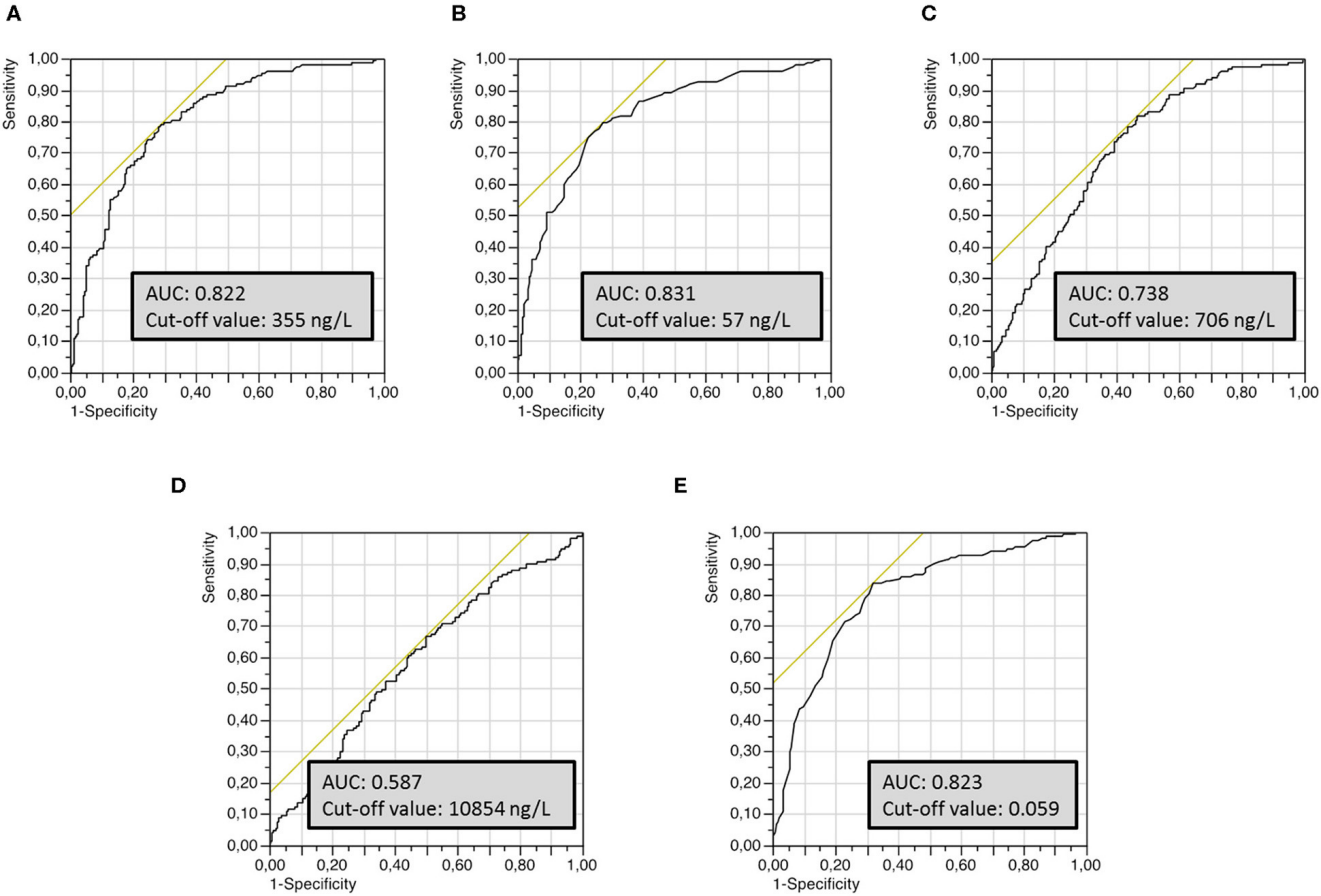
Univariate receiver operating characteristics (ROC) curves of the different CSF biomarkers used to establish optimal cut-off values for the discrimination of AD from non-AD groups. **(A)** T-Tau protein, **(B)** P-Tau_181_ protein, **(C)** Aβ_42_ peptide, **(D)** Aβ_40_ peptide, **(E)** Aβ_42_/Aβ_40_ ratio.

Univariate ROC curves analyses of T-Tau, P-Tau_181_, Aβ_42_, Aβ_40_ concentrations and Aβ_42_/Aβ_40_ ratio levels yielded cut-off values for the diagnosis of AD of ≥355, ≥57, ≤706, ≥10,854, and ≤0.059 ng/L, respectively (Figure 1). Sensitivity, specificity, AUC analysis, positive and negative predictive values for each cut-off value are summarized in Table 2. T-Tau, P-Tau_181_ and Aβ_42_/Aβ_40_ ratio had a high discriminating power between AD and non-AD populations (AUC = 0.822, 0.831, and 0.823, respectively) whereas Aβ_40_ concentration showed the lowest AUC (0.578).

**Table 2:** Optimal cut-off value for each biomarker for the discrimination of AD from non-AD populations, based on AUC analyses of ROC curves and corresponding sensitivity, specificity and predictive values.

	**Cut-off**	**AUC**	**Sensitivity**	**Specificity**	**Predictive**	**Predictive**
	**value**		**(%)**	**(%)**	**value (+)**	**value (–)**
T-Tau	355 ng/L	0.822	81.4	72.5	0.63	0.87
P-Tau_181_	57 ng/L	0.831	82.9	73.3	0.64	0.88
Aβ_42_	706 ng/L	0.738	85.7	54.6	0.52	0.87
Aβ_40_	10854 ng/L	0.587	63.6	53.8	0.45	0.72
Aβ_42_/Aβ_40_ ratio	0.059	0.823	87.1	68.8	0.62	0.90

*AUC, area under the curve*.

In the clinically diagnosed AD population (*n* = 140), 88 (62.9%) of patients had a CSF AD profile by using a classification strategy based solely on the core CSF biomarkers (Tau, P-Tau_181_, Aβ_42_). In the same way, in the non-AD population (*n* = 240), 90 (37.5%) patients had a normal CSF profile and 23 (9.6%) patients had an AD profile (and were misclassified). The remaining patients (37.1% in AD and 52.9% in non-AD populations) had conflicting results of CSF biomarkers.

**Figure 2:**
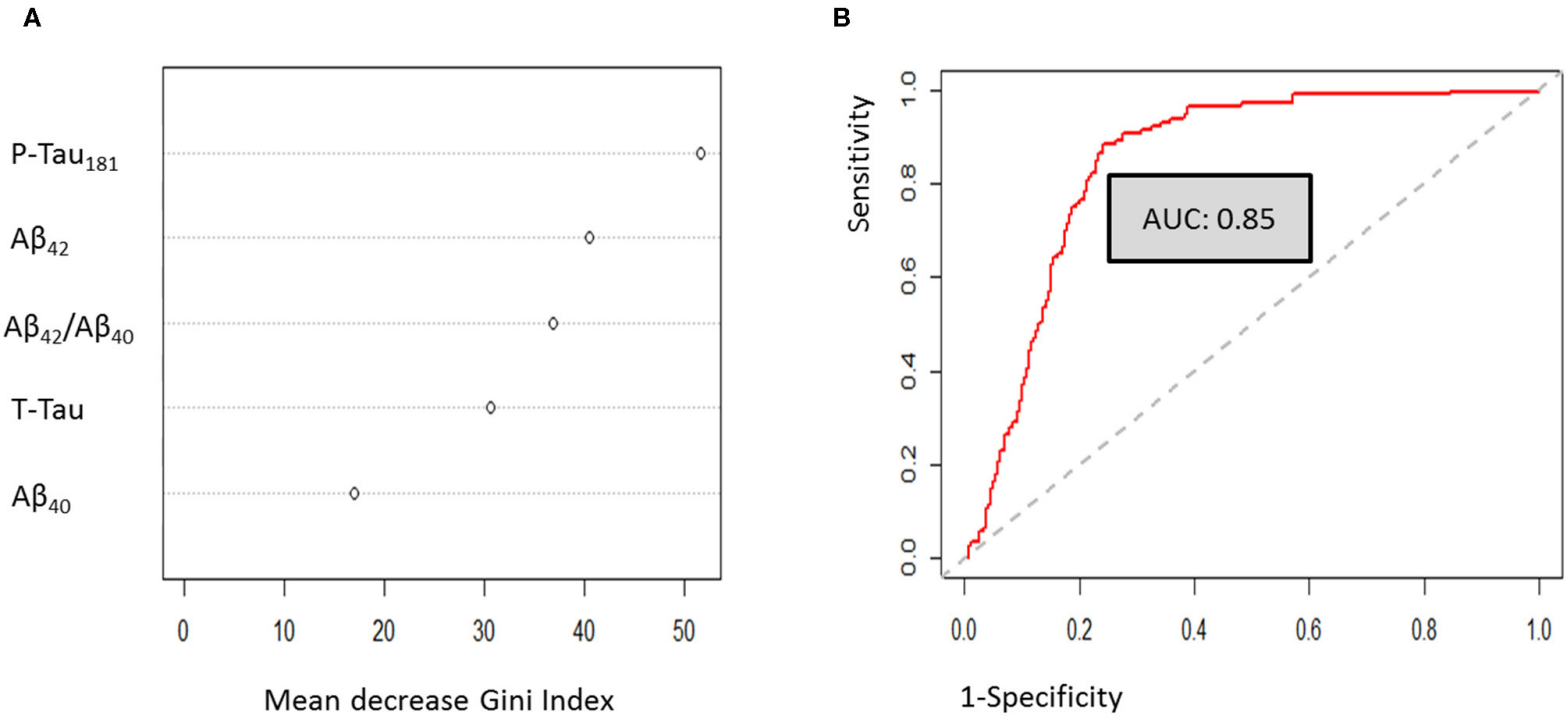
Multivariate analysis using random forest approaches. **(A)** Gini-Index evaluation for the optimized model. **(B)** Random forest ROC curve analysis.

Multivariate analysis using a random forest machine learning approach was used to define the best diagnostic algorithm for the discrimination of AD from non-AD patients. After optimization, Gini-Index showed that P-Tau_181_, Aβ_42_ and Aβ_42_/Aβ_40_ ratio were the CSF biomarkers with the best discriminatory capacity (53.3, 41.9, and 37.6, respectively). By contrast, the impact of T-Tau protein and Aβ_40_ peptide remained lower (27.7 and 16.4 respectively) (Figure 2A). To evaluate the discriminatory capacity of the final random forest model, a ROC curve was plotted, and showed an AUC of 0.85 (Figure 2B). The classification error of random forest (out-of-bag estimate), was about 20%. The sensitivity and specificity of the random forest model were about 76 and 82%, respectively.

**Figure 3:**
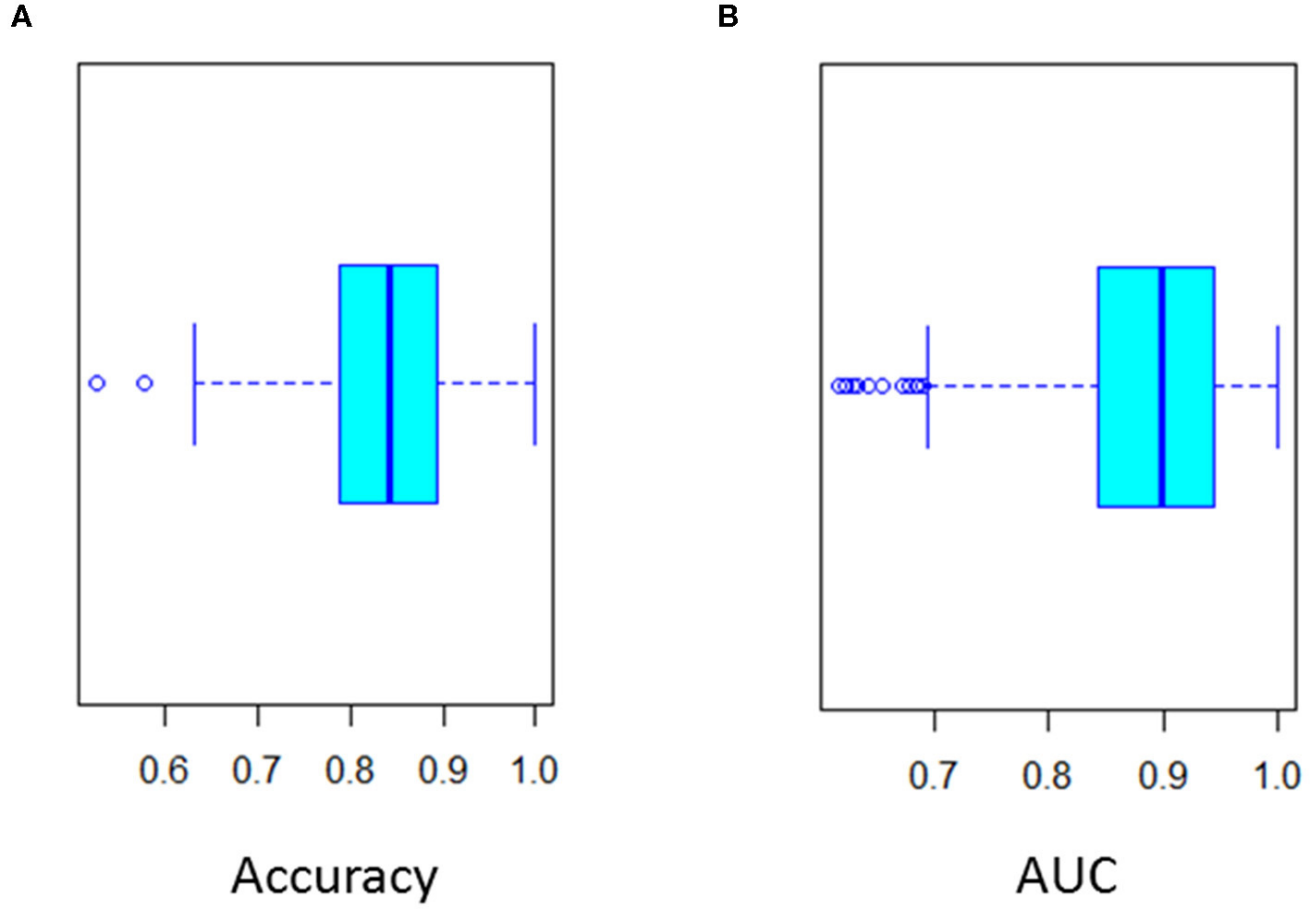
Twenty-fold cross validation of multivariate logistic regression analysis. **(A)** Accuracy (Mean 0.85) and **(B)** ROC curve AUC (Mean 0.89) of the cross validation.

To improve classification of CSF biomarkers in daily practice, a multivariate logistic regression model was constructed. This analysis identified the same discriminating biomarkers as the random forest analysis (namely P-Tau_181_, Aβ_42_, and Aβ_42_/Aβ_40_ ratio) (Table 3). The final model retained by binary logistic regression combining these three main CSF biomarkers was cross-validated 20-fold. Cross validation showed that this model yielded the most discriminatory approach [mean accuracy: 0.83 (0.65 – 1); mean AUC: 0.89 (0.7 – 1)] (Figure 3). This model validated the following equation, combining P-Tau_181_, Aβ_42_ concentrations and Aβ_42_/Aβ_40_ ratio biomarkers, which could be considered in daily practice to discriminate AD from non-AD patients:

$$\begin{array}{l} pAD=\;\frac{1}{1+e^{-Zi}} \end{array}$$

Zi = – 0.5008 + [0.0429 × P-Tau_181_ (ng/L)] – [0.0028 × Aβ_42_ (ng/L)] – [14.675 × Aβ_42_/Aβ_40_ ratio]

**Table 3:** Association between CSF biomarkers and AD as evaluated by univariate analysis and logistic regression.

**Variables**	**AD**	**Non-AD**	**Univariate analysis**	**Multivariate analysis**	
			***p***	***p***	**OR**
T-Tau (ng/L)	617	319	<0.001	NS	
P-Tau_181_ (ng/L)	82	47	<0.001	6.97 × 10^**-**10^	1.044 [1.030**–**1.058]
Aβ_42_ (ng/L)	513	786	<0.001	2.60 × 10^**-**6^	0.997 [0.995**–**0.998]
Aβ_40_ (ng/L)	12 956	11 304	<0.01	NS	
Aβ_42_/Aβ_40_ ratio	0.044	0.077	<0.001	0.04	4.23 × 10^**-**7^ [2.157 × 10^**-**13^**–**0.186]

*AD Alzheimer disease, non-AD various non-AD diagnoses, OR odds ratio*.

*CSF concentrations of T-Tau, P-Tau_181_, Aβ_42_, Aβ_40_ and Aβ_42_/Aβ_40_ ratio were included in multivariate logistic regression analysis*.

pAD was defined as the predictive probability of AD. ROC curve of pAD showed an AUC of 0.89. The optimal cut-off was 0.387 (sensitivity 85% and specificity 85%).

## Discussion

In daily practice, CSF biomarkers are likely to play a key role in the diagnosis of AD, especially in the presence of discrepancies between imaging and clinical features, or atypical presentation of the disease. Nevertheless, the classification of CSF biomarker assay results can be difficult, due to the lack of consensus on clinically relevant thresholds, even when using the same assays. These differences are the combined result of pre-analytical and analytical factors. For example, pre-analytical factors, such as the choice of lumbar puncture needle, collection tube or conservation tube, remain the main confounding factors, although recommendations have been published to promote their standardization (31).

Based on our cohort recruited in memory consultations and geriatrics wards, we firstly defined the optimal cut-off values that can adequately discriminate between AD and non-AD populations using Innotest ELISA methods (Fujirebio Europe, Ghent, Belgium). Recent studies have defined cut-off values using the same ELISAs as those used in our study. For instance, a study using data-drive Gaussian mixture modeling determined a cut-off of 680 ng/L for CSF Aβ_42_ peptide concentration, which is very close to that found in our study (12). In an autopsy-confirmed AD population, the different cut-off values were also consistent with our findings, with the greatest difference observed for Aβ_42_ peptide (638.5 vs. 706.0 ng/L, respectively) (14). P-Tau cut-off value found in our study was consistent with those found in the literature of around 60 ng/L (10). T-Tau protein cut-off value of 500 ng/L was close to the commonly used concentration in daily practice for the elderly population (32). Furthermore, the use of a gray zone in the decisional algorithm has been suggested to assist in the classification based on CSF biomarkers (e.g., +10% for T-Tau and P-Tau_181_ concentrations and−10% for Aβ_42_ concentrations and Aβ_42_/Aβ_40_ ratio) (33), in order to take account of the analytical variability of CSF biomarker assays. Only a few studies have defined a specific cut-off value for classification based on CSF Aβ_40_. For example, Dorey et al. suggested an increased concentration of Aβ_40_ peptide (>12,644 ng/L) in AD compared with non-AD patients. However, this cut-off value was determined in AD patients with Aβ_42_ concentrations above the cut-off value or non-AD patients with decreased Aβ_42_ concentration (11). In our study, all 380 patients were assessed (i.e., all AD and non-AD patients, blinded to the Aβ_42_ concentration) in order to define our optimal cut-off value of 10,854 ng/L. These cut-off values are in agreement with those commonly described in the literature (15, 34).

CSF concentration of Aβ_42_ peptide is one of the most commonly used criteria in the diagnosis of AD, because of its accumulation in amyloid plaques in the brain. However, different studies have demonstrated that CSF concentrations of amyloid peptide (i.e., Aβ_42_ and Aβ_40_) may be influenced by inter-individual variations in the total amyloid load linked to variations in production and/or turnover in the brain (9). A decreased CSF concentration of Aβ_42_ peptide may be the consequence of lower production of all amyloid peptides, or of an accumulation of Aβ_42_ peptide in amyloid plaques in the brain. In case of low levels of production of all amyloid peptides, the concentration of Aβ_40_ peptide will also be decreased, which could lead to profile misclassification. For that purpose, the calculation of the amyloid Aβ_42_/Aβ_40_ ratio has been proposed to differentiate the two situations. The addition of the Aβ_42_/Aβ_40_ ratio to the diagnostic algorithm based on core CSF biomarker analysis improves the diagnosis performance.

Univariate analysis was used to define cut-off for CSF biomarkers, whereas multivariate analysis was preferred to study the most efficient combination of CSF biomarkers for the diagnosis of AD. The discriminatory capacities of T-Tau protein and Aβ_40_ concentrations were clearly weaker than those of P-Tau_181_ and Aβ_42_ concentrations and the Aβ_42_/Aβ_40_ ratio. Our findings are consistent with those of Slaets et al., who reported that the addition of the Aβ_42_/Aβ_40_ ratio to standard CSF biomarkers improved discrimination between AD and non-AD profiles, whereas the addition of Aβ_40_ peptide concentration alone did not (15).

In our cohort, the two multivariate prediction models showed that the algorithm based on P-Tau_181_, Aβ_42_, and Aβ_42_/Aβ_40_ seems to be the most relevant for discriminating AD from non-AD populations. Our results are consistent with the conclusion of a recent review that recommended measuring the Aβ_42_/Aβ_40_ ratio irrespective of the concentration of Aβ_42_ peptide (16). The results previously published by Bombois et al. which concluded that the measurement of CSF Aβ1-42 and p-Tau levels seems sufficient for the diagnosis of AD are also in agreement with our data (35). However, for many years, the standard CSF biomarker analysis strategy in AD has been based on the interpretation of T-Tau, P-Tau_181_, and Aβ_42_ concentrations. Our results suggest that T-Tau protein measurement could be replaced by Aβ_40_ concentration measurement, in order to calculate Aβ_42_/Aβ_40_ ratio. Our results are consistent with the literature which indicates that CSF P-tau should be considered the most specific biomarker for AD (36). Nevertheless, despite a less important impact in the discrimination between AD and non-AD population, T-Tau protein assay remains useful for the evaluation of diseases associated with acute brain injury (37) (e.g., encephalitis, Creutzfeldt-Jakob disease, cerebral infarction). An acute brain injury is associated with neuronal death which releases large amounts of T-Tau protein. The increase in CSF T-Tau protein concentration occurs from the first few days following the injury and lasts for some weeks, whereas P-Tau protein concentration remains normal (37). An increased T-Tau protein concentration may be measured in absence of neurocognitive disorders such as AD. The high rate of comorbidities commonly found in elderly patients may decrease the specificity of T-Tau protein concentration. In absence of control group, these results confirmed that CSF biomarkers are useful in the differential diagnosis of AD vs. other causes of neurocognitive disorders, rather than in the discrimination between AD patients and healthy subjects.

The population of our study was older than those of previous studies. Interestingly, Ewers et al. showed that the specificity and negative predictive value of CSF biomarkers decreased with age (38). As a result, the increased number of false negative results (patients with AD with negative CSF biomarkers) may be highlighted in our relatively older population, which also explains the relatively high proportion of misclassified results compared to the literature. The recently described Limbic-predominant Age-related TDP-43 Encephalopathy (LATE) disease may also explain these results in an old population. Patients with LATE disease often includes tauopathy and amyloïd-β plaques which could mimic Alzheimer's-type dementia (39). Moreover, different confounding factors such as the high rate of mixed dementia in elderly (40) or the discrepancies between neurocognitive disorders and neuropathological lesion of AD at autopsy (41, 42) could decrease the discriminative power of CSF biomarkers.

The main limitation of this study is the lack of neuropathological validation of the diagnosis, which could explain the rate of misinterpreted results in both populations under study, especially the non-AD patients. Nevertheless, we attempted to minimize the rate of probable misdiagnosis by using the most specific and sensitive criteria for each patient in our study. Diagnoses were made by a multidisciplinary team composed of trained physicians (neurologists, geriatricians and psychiatrists). The 5-year follow-up allowed physicians to revise their diagnosis, if an atypical clinical or cognitive sign was detected. A decrease of CSF Aβ_42_ peptide concentration has been reported in DLB patients which may have an effect of non-AD population results, despite the low number of patients with DLB retained diagnosis in our cohort. Clinicians were blinded to CSF biomarker results prior to clinical diagnosis, which excluded a circular reasoning bias in our findings. However, recent reports highlighting that 10% to 30% of patients clinically diagnosed as AD by experts do not display AD neuropathologic changes at autopsy (43). That is why the lack of supporting biomarkers regarding the diagnosis of AD could also be considered a limitation of this study. A further limitation is the single-center nature of the study.

## Conclusion

This study confirms that the Aβ_42_/Aβ_40_ ratio is more useful than the Aβ_40_ concentration alone for discriminating AD from non-AD populations in daily practice.

Both random forest and logistic regression multivariate analysis showed that a diagnostic algorithm based on P-Tau_181_, Aβ_42_, and the Aβ_42_/Aβ_40_ ratio is the most relevant for distinguishing AD from non-AD patients. These results suggest that the Aβ_42_/Aβ_40_ ratio should be calculated in all cases, independently of Aβ_42_ concentration. However, T-Tau protein assay remains useful in daily practice to rule out differential diagnoses.

## Data Availability Statement

The datasets generated for this study are available on request to the corresponding author.

## Ethics Statement

The study was approved by the regional ethics committee (CPP Est III; protocol number 2014-A00056-41), and written informed consent was obtained from each participant and their main caregiver.

## Author Contributions

J-BO, ZD, NP, PG, and RM contributed conception and design of the study and wrote the final version of the manuscript. VN, SB, L-AB, AD, YJ, J-LN, and RM included patients and classified patients in this study. ZD performed the statistical analysis. J-BO, ZD, and RM wrote the first draft of the manuscript. All authors contributed to manuscript revision, read and approved the submitted version.

## Conflict of Interest

The authors declare that the research was conducted in the absence of any commercial or financial relationships that could be construed as a potential conflict of interest.
